# Combined Transcriptomic and Proteomic Analysis of *Myzus persicae*, the Green Peach Aphid, Infected with Cucumber Mosaic Virus

**DOI:** 10.3390/insects12050372

**Published:** 2021-04-21

**Authors:** Yan Liang, Kang-Sheng Ma, Ping-Zhuo Liang, Li-Wen Yang, Lei Zhang, Xi-Wu Gao

**Affiliations:** 1 Department of Entomology, China Agricultural University, Beijing 100193, China; liangyan20061@163.com (Y.L.); kangshengma@mail.hzau.edu.cn (K.-S.M.); liangpingzhuo@126.com (P.-Z.L.); liwenyang1393@163.com (L.-W.Y.); 2 Institute of Plant Health, Beijing Florascape Co., Ltd., Beijing 100032, China

**Keywords:** cucumber mosaic virus (CMV), iTRAQ, *Myzus persicae*, transcriptome

## Abstract

**Simple Summary:**

In this study, an integrated analysis of the mRNA and protein was performed to identify important putative regulators involved in the transmission of CMV (cucumber mosaic virus) by aphids. At the level of transcription, a total of 20,550 genes (≥2-fold expression difference) were identified as being differentially expressed genes (DEGs) 24 h after healthy aphid transfer to infected tobacco plants using the RNA-seq approach. At the protein level, 744 proteins were classified as being differentially abundant between virus-treated and control *Myzus persicae* using iTRAQ (isobaric tags for relative and absolute quantitation) analysis. The combined mRNA and protein analysis enabled the identification of some viral putative regulators, such as cuticle proteins, ribosomal proteins, and cytochrome P450 enzymes. The results show that most of the key putative regulators were highly accumulated at the protein level. Based on those findings, we can speculate that the process by which aphids spread CMV is mainly related to post-translational regulation rather than transcription.

**Abstract:**

Aphids transmit CMV (cucumber mosaic virus) in a non-persistent manner. However, little is known about the mechanism of CMV transmission. In this study, an integrated analysis of the mRNA and protein was performed to identify important putative regulators involved in the transmission of CMV by aphids. At the level of transcription, a total of 20,550 genes (≥2-fold expression difference) were identified as being differentially expressed genes (DEGs) 24 h after healthy aphid transfer to infected tobacco plants using the RNA-seq approach. At the protein level, 744 proteins were classified as being differentially abundant between virus-treated and control *M. persicae* using iTRAQ (isobaric tags for relative and absolute quantitation) analysis. The combined mRNA and protein analysis enabled the identification of some viral putative regulators, such as cuticle proteins, ribosomal proteins, and cytochrome P450 enzymes. The results show that most of the key putative regulators were highly accumulated at the protein level. Based on those findings, we can speculate that the process by which aphids spread CMV is mainly related to post-translational regulation rather than transcription.

## 1. Introduction

The green peach aphid, *Myzus persicae* (Sulzer) (Homoptera: Aphididae), is one of the most important agricultural pests worldwide [1]. Aphids have a wide host range, exceeding 400 plant species from 40 different plant families, and can cause severe yield losses in agricultural production systems [2]. In addition to sucking phloem sap, aphids are also vectors for the transmission of more than 200 different plant viruses [1,3]. In general, aphids live on different hosts in the winter and in the summer. Aphids exhibit parthenogenetic reproduction in some conditions, so they can reproduce on the same host in all seasons, and the harm caused by aphids is enduring. Due to the parthenogenetic reproduction of their hosts, plant viruses spread by aphids may cause outbreaks that can lead to significant crop losses. In addition, plant virus also can affect behavior of aphid vectors. Peñaflor et al. found the population growth of *Aphis glycine* was reduced and the probing preferences was increased on soybean mosaic virus (SMV)-infected soybeans [4]. *A. gossypii* showed an increased number of probes on cucumber mosaic virus (CMV)-infected cucumber [5], which also enhanced the transmission of viruses. Understanding the mechanism of virus transmission by *M. persicae*, therefore, is critical for controlling its occurrence and spread. Knowledge of the mechanism of virus transmission could help us to establish a novel method for protecting crops.

Cucumber mosaic virus (CMV), a typical member of the genus *Cucumovirus* in the family Bromoviridae, is widespread in many countries around the world [6]. CMV has a broad host range, infecting over 1200 species in 100 plant families and is transmitted via seeds, vectors, and friction. The coat protein (CP) 3b, encoded by genomic RNA 3, is known to be related to virus transmission by aphids [7,8,9]. The 2b counter-defense protein, encoded by the 3′-proximal open reading frame of CMV RNA 2, can inhibit plant host resistance to *M. persicae* [10,11]. The 2b protein can also increase the levels of the plant reactive oxygen species (ROS) H_2_O_2_ to enhance CMV acquisition and transmission by *M. persicae* [12]. In addition, it has long been known that CMV does not require helper components to be spread by aphids [13]. Aphids transmit CMV in a non-persistent manner, and in this manner, virus particles bind to putative regulators within the stylet and are released during salivation [14,15].

The mechanism of virus transmission by *M. persicae* is quite complicated and involves a combination of changes in gene transcription and protein. Many genes and proteins are known to participate in this process. Therefore, it is advantageous to concurrently analyze transcriptomic and proteomic results in order to aid in the identification of candidate virus putative regulators in the insect vector and to characterize the metabolic pathways governing the transmission process. Recent reports have documented extraordinary progress in this area using transcriptomic and proteomic analyses [16,17,18,19,20,21]. Laminin subunit alpha, dystroglycan, integrin alpha-PS2, and cuticle proteins are involved in tomato yellow leaf curl virus (TYLCV) and papaya leaf curl China virus (PaLCuCNV) transport by whitefly [22]. Cilia et al. found the quantitative and heritable proteomic variation can lead to the specificity of virus transmission by aphids [23]. Barley yellow dwarf virus-GPV (BYDV-GPV) is transmitted by *Rhopalosiphum padi*. Two methods, namely isobaric tags for relative and absolute quantitation (iTRAQ) and the yeast two-hybrid (YTH) system, were used to identify proteins in *R. padi*. Some proteins related to viral transmission, including glyceraldehyde-3-phosphate dehydrogenase, ATP synthase subunit beta, cuticular protein, peroxiredoxin, and the cuticular protein and proteasome subunit beta, also were identified by Wang et al. [16]. Cyclophilin proteins play an important role in cereal yellow dwarf virus (CYDV)-RPV transmission by *Sitobion graminum*, probably during crossing of the hindgut [24]. However, several studies have shown that the correlations between mRNA and protein levels can be relatively low in different samples [20,25,26,27]. A likely explanation is that the genes involved in post-transcriptional and translational processing show variation in their spatiotemporal expression patterns in different pathways. In the present study, we compared CMV-infected aphids and healthy aphids at both the transcriptomic (RNA-seq) and the proteomic (iTRAQ) levels to expand our knowledge of the mechanism(s) underlying *M. persicae* virus transmission.

## 2. Materials and Methods

### 2.1. Plant and CMV Inoculation

Tobacco (*Nicotiana tabacum* L.) plants were grown in insect-free cages in a glasshouse at 24 °C under artificial light with a 16-h light/8-h dark photoperiod. The samples infected with CMV were collected from a field in Shou Guang, in Shandong province in China. The CMV-SXCH isolate (GenBank NO. JX993913) was propagated in tobacco plants, purified by the method of Ng and Perry, and stored at −80 °C [28].

**Figure 1:**
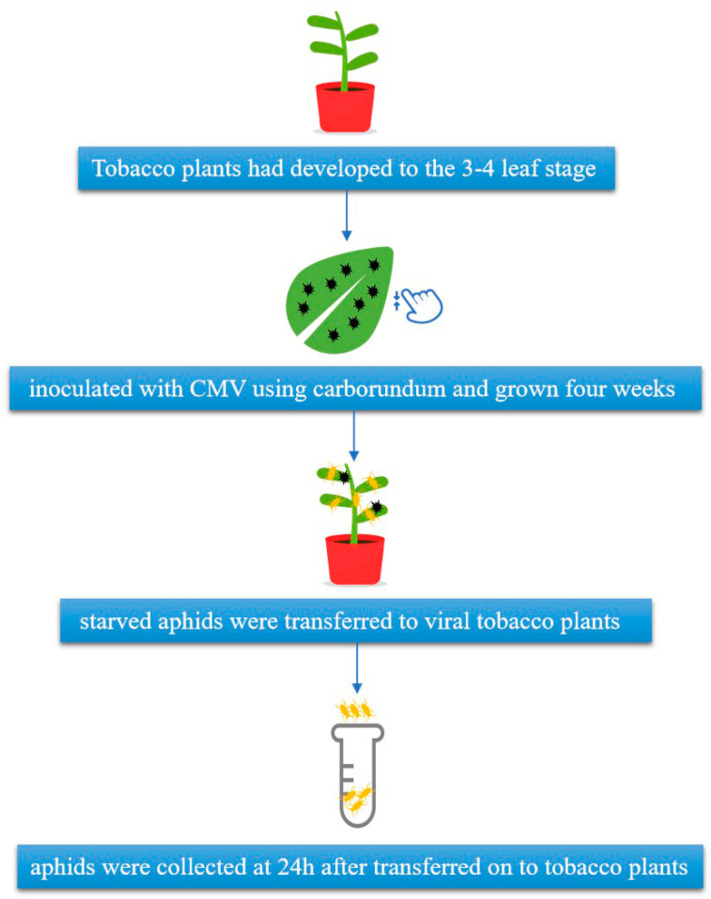
Workflow for sample collection.

When the tobacco plants had developed to the 3–4 leaf stage, they were inoculated with virus using carborundum to abrade the leaf surface. Crude extracts were prepared from CMV-infected tobacco leaf tissues in 0.01 M phosphate buffer (pH 7.0) at a 1:5 (*w*/*v*) ratio. The experimental group was treated with crude leaf extracts, while the mock inoculation (control) experiments used water instead of buffer. All inoculations used the same stock of frozen CMV-infected leaf material. The CMV-inoculated plants were maintained for four weeks to allow them to develop an infection, after which they were used in the experiments (Figure 1).

### 2.2. Aphids and Sample Collection

*M. persicae* (Sulzer) individuals were maintained on healthy tobacco plants in cages. Adult apterous aphids were collected and starved for three hours. The starved aphids were then transferred to tobacco plants that had been inoculated with the virus four weeks previously. Then the control aphids were transferred to healthy tobaccos after being starved. Previous research in our lab has indicated that the peak period for maximum CMV in aphids occurs at 24 h after healthy aphids are transferred to virus-infected tobacco plants (data not shown). Therefore, six aphid samples were collected at 24 h, including control aphids; each sample consisted of ~65 individuals, and three biological replicates were collected. All samples were frozen in liquid nitrogen and stored at −80 °C until use.

### 2.3. RNA Extraction, Nucleotide Sequencing, and Raw Data Processing

Total RNA was extracted from the aphids with TRIzol reagent (Invitrogen, Carlsbad, CA, USA). The quality of the RNA was assessed by measuring the absorbance at 260 nM with a NanoDrop spectrophotometer (Thermo Fisher Scientific, Waltham, MA, USA) and analysis on an Agilent 2100 bioanalyzer (Agilent, Santa Clara, CA, USA). Each RNA sequencing library was constructed from 5 μg of total RNA.

RNA sequencing was performed on an Illumina HiSeq™ 2500 instrument (Illumina, San Diego, CA, USA). Briefly, mRNA was enriched by oligo (dT) affinity purification and randomly fragmented into 200 bp fragments by treatment with metal ions; first-strand cDNA synthesis was then performed after which sequencing adaptors were ligated to the ends of the fragments [29].

In order to assess the quality of the libraries and the sequencing performance, the quality of the raw data was determined. The adapter sequences were first removed from the raw reads, and quality filtration was performed. This mainly involved removing reads with no insert and reads with low quality bases, and discarding reads in which the percentage of Ns (unknown bases) was >10% and also sequences <20 bp in length. The clean data was assembled using Trinity software (version: trinityrnaseq-r, 25 February 2013) to study the transcriptome; no reference genome was used for read alignment.

### 2.4. Identification of Differentially Expressed Genes (DEGs)

Gene expression levels were normalized with fragments per kilobase of exon per million mapped reads (FPKM) values.

(1) $$\mathrm{FPKM}=\frac{\mathrm{cDNA}\;\mathrm{Fragments}}{\mathrm{Mapped}\;\mathrm{Fragments}\times \mathrm{Transcript}\;\mathrm{Length}}$$

cDNA fragments represent the number of fragments that are compared to a transcript; mapped fragments (millions) represent the total number of fragments compared to the transcript, and 10^6^ is one unit; transcript length (kb): 10^3^ is one unit.

The *p*-values presented correspond to a differential gene expression test [26]. The false discovery rate (FDR) approach that we used here is a method to determine the *p*-value threshold in multiple tests [26]. To reduce false positive results, we reported all data based on a *p*-value of <0.05 (95% confidence) and an FDR < 0.1%. For the transcriptome analysis, a 2-fold cutoff value was used as the criterion for the identification of both up-regulated and down-regulated genes.

### 2.5. Protein Extraction, Digestion, and iTRAQ Labeling

The aphid samples used for iTRAQ analysis were the same as those used for transcriptome sequencing. The method of protein extraction was that reported previously by Wei et al. [30]. In brief, samples were suspended in extraction buffer (10% (*w*/*v*) trichloroacetic acid/acetone solution containing 65 mM dithiothreitol (DTT)), Tris-HCl was added, the samples were centrifuged, and yield of the supernatant was recovered. The proteins were precipitated by the addition of ammonium sulfate-saturated methanol and incubation overnight at −20 °C. After centrifugation and removal of the upper phase, the extracted proteins were washed twice using cold acetone, air-dried, and then finally dissolved in lysis buffer (7 M urea, 2 M thiourea, 0.1% CHAPS). The total protein concentration was measured using the Bradford method.

Two-hundred micrograms of total protein from each sample was used for the protein digestion. First, the volume of the protein solution was adjusted to 125 µL by adding 8M UA (8 M urea, 150 mM Tris-HCl, pH 8.0). After adding 5 μL DTT (200 Mm, Bio-Rad, Hercules, CA, USA), each sample was incubated for 1 h at 37 °C and supplemented with 10 µL IAA (iodoacetamide, 500 Mm, Bio-Rad) for 1 h at room temperature in the dark. Proteins were precipitated by ice-cold acetone and then were re-dissolved in 100 μL triethylammonium bicarbonate (TEAB, 100 mM) [30]. Trypsin was then added to a final protein/enzyme ratio of 50–100:1 (*w*/*w*). The digestion reaction was incubated overnight at 37 °C. Finally, the digested peptides were precipitated and dissolved in 50 µL dissolution buffer (Applied Biosystems, Foster, CA, USA).

The six samples were then labeled using an iTRAQ Reagent-8 plex Multiplex Kit according to the manufacturer’s instructions (Applied Biosystems). The three virus-inoculated samples were labeled with iTRAQ tags 118, 119, and 121, while the three control samples were labeled with tags 115, 116, and 117.

### 2.6. Reverse Phase Separation and Nano-Liquid Chromatography-Coupled MS/MS

The methods and procedures of quantitative proteomics analyses followed Zhong et al. [17]. After iTRAQ labeling, the samples were mixed and lyophilized by vacuum centrifugation. The pooled mixtures, used for reverse phase separation, were fractionated by strong cationic exchange (SCX) chromatography with a polysulfoethyl column (4.6 × 100 mm, 5 μm, 200 Å, PolyLC Inc., Columbia, MA, USA). The labeled samples were re-suspended in 150 μL of buffer A (10 mM KH_2_PO_4_ pH 3.0, 25% acetonitrile (ACN)). The peptide mix was then fractionated over a 65 min gradient using buffer B (10 mM KH_2_PO_4_, 1 M KCl in 25% ACN; pH 3.0) with a flow rate of 0.8 mL/min. The gradient was 0–10% buffer B for 7 min, 10–20% buffer B for 10 min, 20–45% buffer B for 15 min, and 45–100% buffer B for 5 min. Forty-eight fractions were collected, dried, and desalted on C18 spin columns (Thermo Fisher Scientific, Waltham, MA, USA). Finally, the 48 fractions were merged into 10 fractions which were re-dissolved in 30 μL of 0.1% formic acid in 2% ACN and analyzed via LC–MS/MS.

LC-MS/MS analysis was performed with an Orbitrap Velos Nano analyzer (Thermo). The samples were loaded on a trap column (EASY column 5 μm-C18, 200 mm × 100 μm, Thermo Fisher Scientific, Foster, CA, USA) using solvent A (0.1% formic acid). After desalting, the samples were switched online with the analytical column (EASY column 3 μm-C18, 75 μm × 100 mm, Thermo Fisher Scientific, Foster, CA, USA) using solvent B (84% acetonitrile and 0.1% formic acid). Then the samples were fractionated at a flow rate of 2 μL/min for 15 min, and then eluted at 300 nL/min for 101 min. The gradient was 0–35% B for 100 min and 35–100% B for 8 min, and then kept for 12 min.

The methods of data analyses and parameters followed those of [31]. The raw data files were analyzed with the software ProteinPilot. Its parameters were as follows: sample type: iTRAQ 8 plex (peptide labelled); cys alkylation: MMTS; digestion: trypsin; search effort: thorough ID. The MASCOT database was used for searching peak lists. Its parameters were as follows: enzyme: trypsin; fixed modifications: iTRAQ 8 plex (N-term), iTRAQ 8 plex (K), and carbamidomethyl (C); MS peptide tolerance: 10 ppm; MS/MS tolerance: 0.1 Da; number of missed cleavages: up to 1. A peptide list from MASCOT was generated with a false discovery rate < 1%, determined using a concatenated reverse sequence decoy database. Proteins were found using both search algorithms with a minimum of 2 peptides.

### 2.7. Protein Quantification and Correlation Analysis

For protein quantization, a protein must contain at least two unique peptides. The quantitative protein ratios were weighted and normalized by the median ratio in Mascot. Student’s *t*-test was used to analyze the differential expression of proteins. A 1.5-fold cutoff value was the threshold used to characterize the significance of differences in protein expression. To reduce false positive results, we reported all data based on a *p*-value of <0.05 (95% confidence) and an FDR < 0.1%.

Correlation between protein and mRNA expression was performed by Pearson’s chi-squared test with Yates’ continuity correction [32]. According to the standard of differentially expressed genes and proteins, DEGs and DEPs were selected into a searchable database. Then we queried the same expression pattern between the DEGs and DEPs.

### 2.8. Functional and Pathway Enrichment Analysis

Gene annotation was performed using the Blast2GO (http://www.blast2go.com/b2ghome) (accessed on 29 March 2021) program. GO annotation contains biological processes, involved cell components, and molecular functions. The biological interpretation of the differential genes and proteins were further investigated by assigning them to pathways using the Kyoto Encyclopedia of Genes and Genomes (KEGG) annotation tools (http://www.genome.jp/kegg/) (accessed on 29 March 2021) [33]. KEGG pathway and GO enrichment analysis of the differentially expressed proteins were performed, and the formula used was

(2) $$P=\;1\;-\;\sum_{i=0}^{m-1}\frac{\left(\begin{array}{l} M\\ i \end{array}\right)\left(\begin{array}{l} N-M\\ n-i \end{array}\right)}{\left(\begin{array}{l} N\\ n \end{array}\right)}$$

where *N* represents the number of all identified proteins with a GO or a KEGG pathway annotation; *n* is the number of differential proteins in *N*; *M* is the number of proteins that are annotated to the specific GO term or pathway; and m is the number of differential proteins in *M*. If the *p*-value is below 0.05, the GO term or pathway was defined as a significant enrichment of differential proteins. The false discovery rate (FDR) was controlled by the Bonferroni step-down test to correct the *p*-value.

### 2.9. Quantitative Real-Time PCR (qRT-PCR)

A total of 1 μg of RNA was used as the template for first-strand cDNA synthesis using a PrimeScript kit (Takara Biotechnology, Dalian, China) according to the manufacturer’s instructions. Gene-specific primers were designed with DNAMAN 3.0 software. The 18S rRNA gene (GenBank No. AF487716) was used as an internal reference for relative gene expression analysis. The sequences of the primers used in the qRT-PCR analysis are given in Supplementary Table S1. PCR amplification was performed in 20 μL reactions containing 10 μL SYBR^®^ Green real-time PCR Master Mix (Invitrogen), 1 μL of each primer (10 μM), and 1 μL cDNA using an ABI 7500 real-time PCR system (ABI). The thermal cycling conditions were 95 °C for 2 min, followed by 40 cycles of 95 °C for 15 s, 60 °C for 30 s, and 72 °C for 30 s. The relative gene expression levels were calculated using the 2^−ΔΔCT^ method of Livak and Schmittgen [34]. The qRT-PCR data were compared with the corresponding RNA-seq values using Pearson product-moment correlation coefficients. Data were expressed as the mean ± SD of three independent biological replicates.

## 3. Results

### 3.1. Transcriptome Difference Analysis

Using a 2-fold cutoff value as the criterion for identifying up-regulated and down-regulated genes, we identified a total of 20,550 genes as being differentially expressed between the treatment and control aphid groups at 24 h after transfer to tobacco plants. Of these DEGs, 9732 were up-regulated and 10,818 were down-regulated (Supplementary Table S2). The magnitude of the expression differences for the majority of these DEGs (17292, 84%) was between 2- and 5-fold. In the up-regulated genes, c50186, c37331, and c43604 showed the highest fold-changes; in the down-regulated genes, c16109, c3396, and c49381 had the lowest fold-changes (Supplementary Table S3).

**Figure 2:**
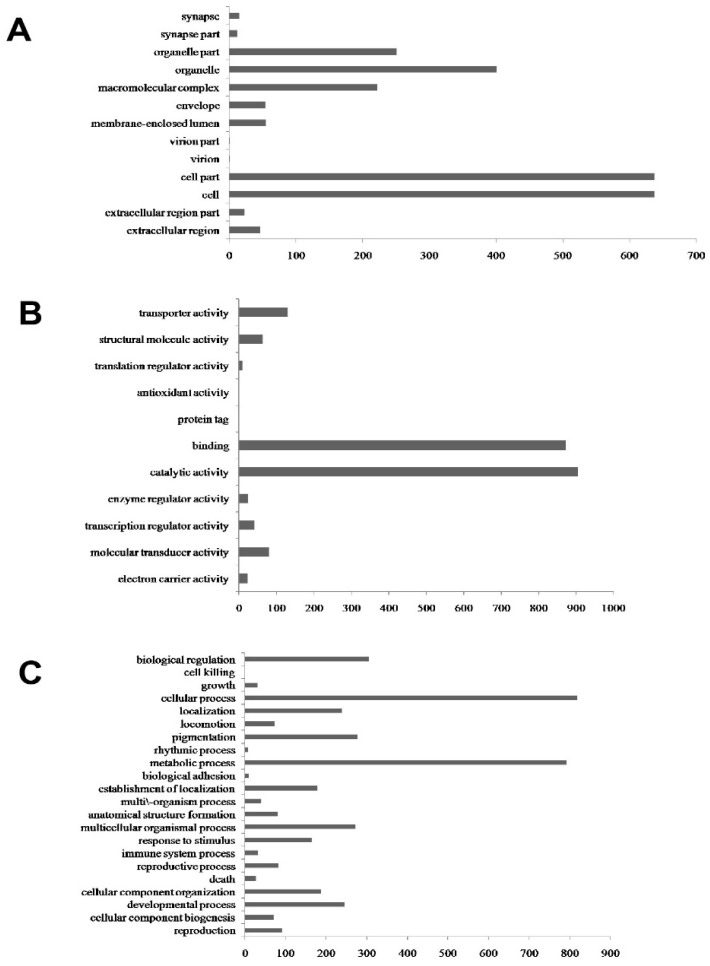
Functional categorization of DEGs between virus-treated and control *M. persicae*. The genes were categorized based on GO annotation using Blast2GO, and the number of DEGs in each category is shown on the x-axes. The three main ontology categories are Cellular Component (**A**), Molecular Function (**B**), and Biological Process (**C**).

Gene Ontology (GO) analysis was performed on the DEGs using Blast2GO (Table S4). Figure 2 shows the distributions of the GO terms in the three main ontology categories, namely “Cellular Component”, “Molecular Function”, and “Biological Process”. “Cell” (637 DEGs), and “organelle” (401 DEGs) were the major terms annotated under Cellular Component (Figure 2A). “Catalytic activity” (904 DEGs), “binding” (874 DEGs), and “transporter activity” (131 DEGs) were the major terms annotated under Molecular Function (Figure 2B). “Cellular process” (818 DEGs), “metabolic process” (792 DEGs), and “biological regulation” (305 DEGs) were the major terms annotated under Biological Process (Figure 2C).

KEGG pathway analysis was performed to identify the main biological pathways affected when aphids are infected with CMV. In the KEGG database, the DEGs were mapped to 501 pathways (Table S5). Among these, 32 pathways were substantially enriched (*p*-value ≤ 0.05) between the up-regulated and the down-regulated DEGs. Examples of enriched pathways include the “cAMP signaling” pathway and “oxidative phosphorylation” (Table 1). Interestingly, the number of up-regulated pathways (25) was far larger than the number of down-regulated pathways (7). It is noteworthy that 46 genes in the “drug metabolism” pathway were enriched, including 22 DEGs annotated as cytochrome P450s and 24 DEGs annotated as other enzymes among the up-regulated genes. In addition to the drug metabolism pathway, there were also 23 DEGs involved in the “metabolism of xenobiotics by cytochrome P450s”. There were 29 DEGs in the “starch and sucrose metabolism” pathway, 13 DEGs in the “galactose metabolism” pathway, and 24 DEGs in the “pentose and glucuronate interconversions” pathway. Some genes were related to the “antigen processing and presentation” pathway. There were also 17 DEGs in the “calcium signaling” pathway. Furthermore, we identified four DEGs related to the “ubiquinone” and “other terpenoid–quinone biosynthesis” pathways. Unexpectedly, only seven DEGs were identified that were predicted to be involved in the “cutin, suberine, and wax biosynthesis” pathway, and all of these genes were down-regulated.

In previous studies, it has been shown that genes related to cuticle proteins, laminin proteins, and ribosomal proteins play important roles in the transmission of viruses by aphids. Our analysis of the RNA-seq data reveals that some of these genes showed dramatically altered expression between the CMV-infected and healthy aphids, especially the genes encoding cuticle proteins and ribosomal proteins (Table 2). As shown in Table 2, 11 DEGs were identified that were annotated as cuticle proteins, and most of these genes were up-regulated. Notably, four DEGs (c567, c31594, c65979, and c36838) among the 11 predicted cuticle protein genes had R&R consensus chitin-binding domains. Another two cuticle protein genes (c66065 and c78688) were down-regulated in the DEGs, and were predicted to function as precursors in the synthesis of cuticle proteins. In addition, 13 DEGs were annotated as encoding ribosomal proteins, and these genes displayed different expression patterns; nine DEGs were up-regulated and four DEGs were down-regulated.

### 3.2. Validation of the DEGs

**Figure 3:**
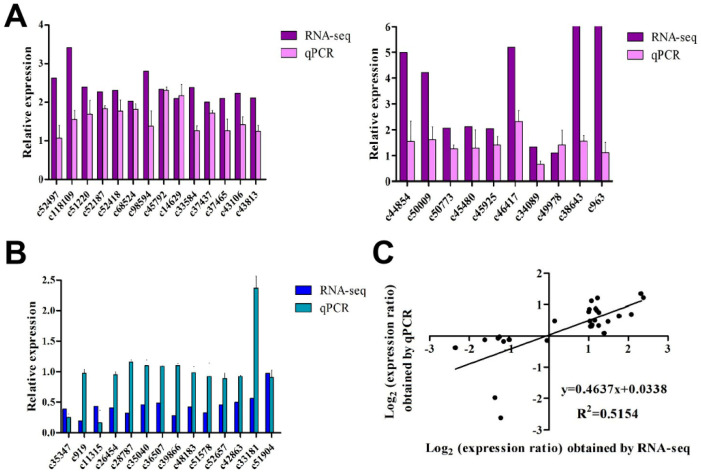
qRT-PCR validation of differential gene expression. (**A**–**C**) Transcript levels for 38 genes, of which 24 were up-regulated (**A**) and 14 were down-regulated (**B**), based on the RNA-seq and qRT-PCR expression data. (**C**) Comparison of the log-transformed gene expression ratios obtained from the RNA-seq data and qRT-PCR data for 38 DEGs.

Real-time quantitative PCR was performed to verify the results of the RNA-seq and proteomic analyses in three biological replicates. A total of 38 genes were selected (Supplementary Table S1) for qRT-PCR analysis (Figure 3). Among the genes tested, the relative expression results for 84% were consistent with those determined from the transcriptome data (32) (Supplementary Table S6). In addition, linear regression analysis showed an overall correlation coefficient of R^2^ = 0.5154, which further indicated a good correlation between the qRT-PCR and RNA-seq data (Figure 3C).

### 3.3. Proteomics Analysis

In this study, we used a 1.5-fold cut-off to select proteins that showed changes in abundance between the sample groups. Using this criterion, a total of 744 proteins were classified as being differentially accumulated between the virus-infected and healthy *M. persicae* groups. Of these proteins, 437 were up-regulated and 307 were down-regulated. The number of up-regulated proteins was greater than the number of down-regulated proteins, which was not consistent with the RNA-seq results. However, a majority of the down-regulated proteins (260) showed relative changes of 1.5- to 2.0-fold (Supplementary Table S7). This result also differed from the transcriptomic data. Notably, some proteins that are related to viral transmission by aphids were found to be down-regulated, including a tentative cuticle protein (3.12-fold), RR2 cuticle protein 3 (1.69-fold), and cuticle protein 4 (1.54-fold). However, some proteins that were related to cytochromes were up-regulated, including cytochrome P450 CYP6CY3 (2.84-fold) and NADPH-cytochrome P450 reductase (2.77-fold). In addition, ribosomal protein L15 was found to be up-regulated by 2.77-fold (Table 3).

**Figure 4:**
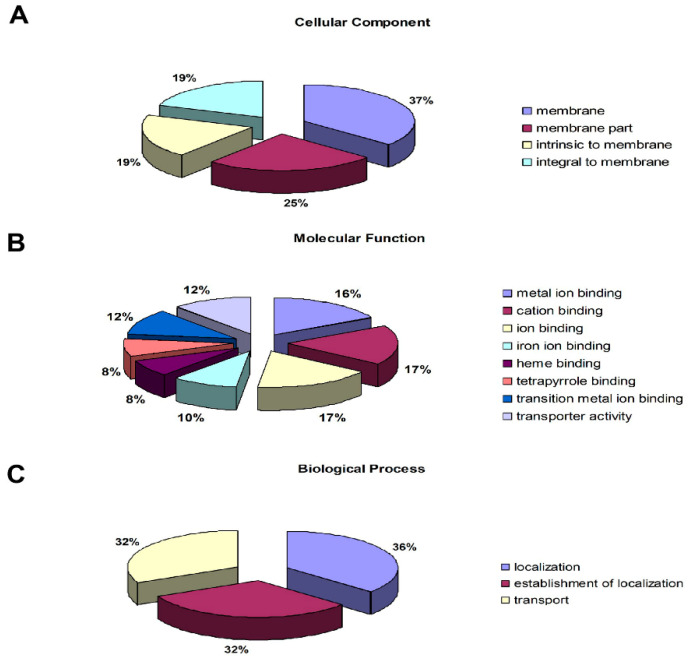
Functional categorization of the differentially-accumulated proteins between virus-infected and control *M. persicae*. The proteins were grouped based on GO annotations, and the percentages of proteins in each GO term are displayed for the Cellular Component (**A**), Molecular Function (**B**), and Biological Process (**C**) categories.

To obtain functional information about the 744 proteins (Table S8), we identified those that showed differential accumulation, and the Cellular Component, Molecular Function, and Biological Process GO categories were queried using the Blast2GO program. The results of the analysis for Cellular Component showed that “membrane” (37%), “membrane part” (26%), “intrinsic to membrane” (19%), and “integral to membrane” (19%) were the top four protein terms (Figure 4A). “Cation binding” (17%), “ion binding” (17%), and “metal ion binding” (16%) were the most abundant terms in Molecular Function (Figure 4B). For the Biological Process category, the differentially accumulated proteins were distributed in the terms “response to localization” (36%), “establishment of localization” (32%), and “transport” (32%) (Figure 4C). These results showed that the distributions of the differentially-accumulated proteins are consistent with the annotation of the DEGs in the Cellular Component, Molecular Function, and Biological Process GO categories (Figure 2). To investigate which biological pathways may be involved when aphids are exposed to virus-infected tobacco plants, the 744 differentially-accumulated proteins were assigned to the KEGG reference pathways. We found that 14 pathways were enriched (*p*-value ≤ 0.05), including “fatty acid metabolism”, “carbon metabolism”, and “amino acid metabolism” (Table 4). The “valine, leucine, and isoleucine degradation” pathway had the lowest *p*-value. The “cytochrome P450” and “cutin, suberine, and wax biosynthesis” pathways, however, were not in the *M. persicae* proteome KEGG database (*p*-value ≤ 0.05). The results of the KEGG database analyses differed between the transcriptomic and proteomic data sets (Table 1).

### 3.4. Analysis of the Correlation between the Transcriptome and Proteome

**Figure 5:**
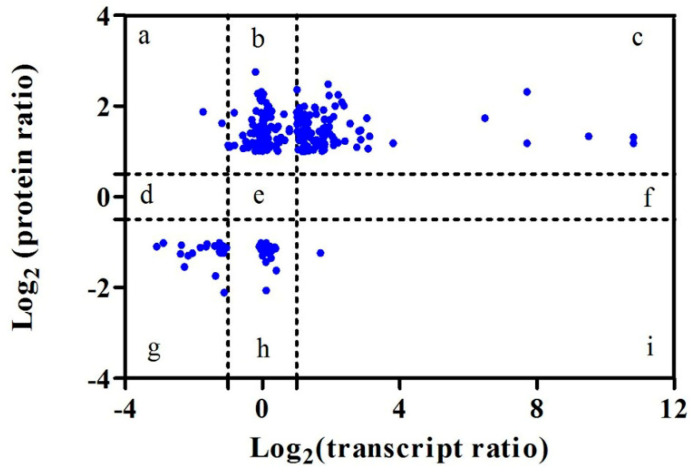
Comparisons of changes in mRNA levels and protein abundance. The relative changes are shown on a log_2_ scale (transcript/protein ratio) on the x- and y-axes, respectively. Quadrant a: the DEGs were down-regulated ≥ 2-fold and the DAPs were up-regulated ≥ 1.5-fold. Quadrant b: DEGs were up-/down-regulated ≤ 2-fold, and DAPs were up-regulated ≥ 1.5-fold. Quadrant c: DEGs were up-regulated ≥ 2-fold and DAPs were up-regulated ≥ 1.5-fold. Quadrant g: DEGs were down-regulated ≥ 2-fold and DAPs were down-regulated ≥ 1.5-fold. Quadrant h: DEGs were up-/down-regulated ≤ 2-fold and DAPs were down-regulated ≥ 1.5-fold. Quadrant i: DEGs were up-regulated ≥ 2-fold and DAPs were down-regulated ≥ 1.5-fold.

Of the 744 identified differentially-accumulated proteins, 282 had corresponding transcripts in the RNA-seq data. Specifically, 207 of these showed the same direction of change (up- or down-regulation) in the two “omics” data sets (Table 5). As shown in Figure 5, almost half of the mRNA:protein ratios (127) were concentrated in quadrants c and g, reflecting significant changes in both the transcript and the protein levels (Supplementary Table S9). Moreover, a few important proteins fell in these two quadrants, including cytochrome P450 CYP6CY3 (c118109), cytochrome c oxidase subunit 2 (c49464), ribosomal protein L15 (c75408), protein BCCIP homolog (c41961), and ATP synthase subunit alpha (c11315). Notably, only two up-regulated proteins, which were both down-regulated DEGs at the mRNA level (quadrant a), were detected; these are AP-3 complex subunit delta (c36095) and an uncharacterized protein (c53987). However, only one putative structural protein (c49848) fell in quadrant i. It was up-regulated in the DEGs, but down-regulated with respect to protein abundance. In total, 45% of the mRNA:protein ratios were found to fall in quadrants c and g (Figure 5), where the ratios reflected significant changes at both the mRNA and the protein levels. This result implies that the mRNA and protein levels were not well correlated.

It is widely known that the cytochrome P450 monooxygenase (P450) system is involved in the detoxification of xenobiotics [35]. The CYP6CY3 gene is associated with resistance to neonicotinoid insecticides in *M. persicae* [36,37]. The NADPH-cytochrome P450 reductase (CPR) gene is considered a vital part of P450-mediated insecticide resistance and is considered a novel target for the development of “smart” insecticides and synergists [38,39]. In this study, cytochrome P450 CYP6CY3 was highly significant changed in the transcriptome and proteome and was up-regulated in two stages, while NADPH-cytochrome P450 reductase was down-regulated in the transcriptome and up-regulated in the proteome. These results suggested that when *M. persicae* was infected by CMV, the CPR gene was consumed to compound the cytochrome related genes in the transcription stage.

## 4. Discussion

The way in which aphids acquire and spread viruses is complicated, and cooperation between genes and proteins at the mRNA and protein levels is necessary for these processes to occur. The main pathways that these proteins and their genes are associated with will be discussed in the following passages.

In non-circulative transmission, cuticle proteins (CuPs) identified in insect stylets play important roles as virus putative regulators [40]. Several previous studies have shown that CuPs are involved in non-circulative virus transmission [41,42,43,44]. Four *M. persicae* cuticular proteins (MpCuPs), including MPCP2, MPCP3, MPCP4, and MPCP5, were identified by Dombrovsky et al. [44]. Two of four MpCuPs, namely MPCP2 and MPCP3, contain the characteristic RR-2 chitin binding domain, while the other two MpCuPs, MPCP4 and MPCP5, have the RR-1 consensus sequence [41]. R&R regions (RR-1 and RR-2) have been found to contain a partially conserved domain in the CuPs [45,46]. In our study, we found that different R&R domain protein genes displayed complex expression patterns. The changes in RR-1 and RR-2 differed at the transcript and protein levels. Specifically, four RR-1-domain protein genes (c567, c31594, c65979, and c36838) were up-regulated at the transcript level (Table 2). One RR-2-domain cuticle protein (Q45V97) was found to be down-regulated at the protein level (Table 3). These results suggest that the two may play different roles in virus transmission. RR-1 plays a role at the mRNA level, while RR-2 plays a role in the protein level. A previous report showed that RR-1 and RR-2 are involved in zucchini yellow mosaic virus (ZYMV) transmission [41]. Two recent studies have shown that the CMV CP (coat protein) interacts with both RR-1 proteins in yeast and with RR-2 peptides in vitro [41,43]. Based on the above results, we suggest that the R&R consensus regions play a critical role in virus spread by insect vectors. However, additional investigations are needed to determine the role of the R&R domains, and which domain in cuticle proteins is necessary for CMV spread.

Recently, the ribosomal protein S2 (RPS2) was identified as a new viral receptor [47]. Prior to this finding, several studies showed that RPS2 shares homology with the laminin receptor precursor, which is known to act as a receptor for several viruses [26,48]. Further investigation found that RPS2 interacts with HC-Pro of tobacco etch virus (TEV). The specific interaction between RPS2 and TEV HC-Pro showed that RPS2 recognizes HC-Pro in the TEV transmission process [47]. In our study, we identified many genes that are related to ribosomal proteins (Table 2), including ribosomal protein S2 (c27431). The genes encoding these ribosomal proteins showed different expression patterns, and 69% were up-regulated. Notably, ribosomal protein L15 (c75408) was up-regulated at both the mRNA and the protein levels. On the contrary, ribosomal protein S2 did not show significantly-different accumulation at the protein level. In the future, investigations should focus on determining whether ribosomal protein L15 can interact with CMV CP in vivo.

Cytochrome P450s, as a family of detoxification enzymes, are involved in the catabolism of various classes of insecticides. Previously-published studies have shown that the CYP6 family of cytochrome P450s is related to insecticide resistance [36,49]. In this study, we were surprised to find that cytochrome P450 CYP6CY3 (c118109) was strongly up-regulated at both the mRNA and protein levels in the bodies of CMV-infected aphids (Table 5). However, potentially important roles of cytochrome P450s in viral transmission have not been reported to date. In addition, cytochrome c oxidase subunit 2 (c49464) had a similar expression pattern to c118109, although cytochrome c oxidase subunit 2 showed much higher expression at the mRNA level than at the protein level. However, NADPH-cytochrome P450 reductase (c51904) displayed a mixed expression pattern; gene expression was down-regulated, but the protein showed increased accumulation. NADPH-cytochrome P450 reductase (c51904) is a substrate for synthesis of cytochrome P450 CYP6CY3 (c118109), so when c118109 was largely synthesized at the mRNA stage, NADPH was highly down-regulated at the same stage. Compared with healthy aphids, both cytochrome P450 *CYP6CY3* (c118109) and cytochrome c oxidase subunit 2 (c49464) were up-regulated at the mRNA level in virus-infected aphids. The increased expression level of P450 and other detoxification enzyme genes were also observed in *Ae. Aegypti* response to ZIKV Infection [50]. These phenomena imply that viruses infection may lead to symptoms similar to insecticides spray such as up regulation of detoxification enzyme genes; therefore, viruliferous aphids synthesize much more cytochrome P450s and Cytochrome c oxidase proteins to deal with the invading CMV.

Viruses that access the stylets of aphids need signaling factors to rapidly recognize the putative regulators. In this study, the signal recognition particle subunit SRP72 (c30589) was strongly up-regulated at the protein level (Table 5), indicating SRP plays an important role in infected aphids that may related to translation and sortation of viral protein [51]. The process of viral recognition of putative regulators also requires energy, including ATP and amino acids. In addition to SRP72, the amino acid transporter (c13794) was up-regulated and showed increased accumulation at the protein level. We imply that the process by aphids recognize viruses maybe mainly influenced by post-translational processing, not by transcription.

Recent studies using transcriptomic and proteomic data have shown that there is a relationship between mRNA levels and protein accumulation [52,53]. However, mRNA expression and protein levels do not always correlate [20,25,27,31]. It is well known that protein levels are largely determined by translational and post-translational processes, and that selective mRNA translation and protein turnover may contribute to the dynamic proteome [54]. In addition, mRNA instability, mRNA-ribosome binding, and protein degradation may lead to the low correlation [55,56]. Therefore, independent analyses of transcriptomic and proteomic data are incomplete, and the two complement one another [20]. However, the extent to which this occurs is still poorly understood. In this study, we performed a comparative analysis of the genetic regulation of the transcriptome and the proteome. As shown in Figure 5, there was a modest relationship between protein accumulation and mRNA expression (R = 0.696). Our results complement the data previously reported for phytoplasma-infected jujube plants, and diapause in the parasitoid wasp *Aphidius gifuensis* Ashmead, showing similar modest protein-transcript correlations [19,20]. Compared to *Bemisia tabaci*, the silverleaf whitefly, our results showed a slightly higher estimate of protein-transcript concordance (correlation coefficients of 0.696 vs. 0.664) when considering differentially accumulated proteins (DEPs) [32].

## 5. Conclusions

In the present study, we compared CMV-infected aphids and healthy aphids at both the transcriptomic (RNA-seq) and the proteomic (iTRAQ) levels to expand our knowledge of the mechanism(s) underlying *M. persicae* virus transmission. The combined mRNA and protein analysis enabled the identification of some viral putative regulators, such as cuticle proteins, ribosomal proteins, and cytochrome P450 enzymes. The results show that most of the key putative regulators were highly accumulated at the protein level. Based on those findings, we can speculate that the process by which aphids spread CMV is mainly related to post-translational regulation rather than transcription.

## Figures and Tables

**Table 1:** Significantly enriched KEGG pathways in the transcriptome of *M. persicae* adults infected with cucumber mosaic virus (CMV).

Pathway	Number of Genes		*p*-Value	Pathway ID
	DEGs ^†^	Expressed ^‡^		
**Up-Regulated**				
Antigen processing and presentation	25	156	6.68 × 10^−5^	ko04612
cAMP signaling pathway	32	270	0.001033478	ko04024
Lysosome	26	225	0.003930307	ko04142
Vasopressin-regulated water reabsorption	12	73	0.004136451	ko04962
Steroid hormone biosynthesis	22	183	0.005010783	ko00140
Metabolism of xenobiotics by cytochrome P450	23	196	0.005434029	ko00980
Bile secretion	19	151	0.005667970	ko04976
Retinol metabolism	21	175	0.006122107	ko00830
Drug metabolism—other enzymes	24	211	0.006467250	ko00983
Chemical carcinogenesis	23	200	0.006738707	ko05204
Legionellosis	15	111	0.007453879	ko05134
Drug metabolism—cytochrome P450	22	192	0.008218267	ko00982
Starch and sucrose metabolism	29	277	0.008386653	ko00500
Pentose and glucuronate interconversions	24	218	0.009174987	ko00040
Ascorbate and aldarate metabolism	21	184	0.010028922	ko00053
Porphyrin and chlorophyll metabolism	22	202	0.013560873	ko00860
Hematopoietic cell lineage	10	70	0.01909453	ko04640
Galactose metabolism	13	103	0.01942347	ko00052
Measles	15	131	0.0259525	ko05162
ABC transporters	18	168	0.027132284	ko02010
Calcium signaling pathway	17	156	0.027367424	ko04020
Ubiquinone and other terpenoid–quinone biosynthesis	4	17	0.03233652	ko00130
Fatty acid biosynthesis	9	70	0.042834788	ko00061
Renin–angiotensin system	12	107	0.048633847	ko04614
Jak-STAT signaling pathway	9	72	0.048990908	ko04630
**Down-Regulated**				
Renin–angiotensin system	13	107	0.004147531	ko04614
Vitamin digestion and absorption	13	112	0.00585761	ko04977
Oxidative phosphorylation	34	431	0.00730206	ko00190
Nicotine addiction	6	32	0.00796758	ko05033
Hypertrophic cardiomyopathy (HCM)	11	91	0.00827137	ko05410
Cardiac muscle contraction	17	192	0.01963673	ko04260
Cutin, suberine, and wax biosynthesis	7	57	0.02967602	ko00073

^†^ The number of differentially expressed genes that belong to each KEGG pathway. ^‡^ The number of expressed genes that belong to each KEGG pathway.

**Table 2:** DEGs that are related to genes encoding cuticle proteins and ribosomal proteins.

Gene ID	log_2_ Ratio	Annotation
**Cuticle Protein-Related Genes**		
c567	1.00	RR1 cuticle protein 2 (*Myzus persicae*)
c31594	1.12	RR1 cuticle protein 7 (*Acyrthosiphon pisum*)
c14422	1.32	cuticle protein (*Lipaphis erysimi*)
c13658	1.79	cuticular protein 70 (*Acyrthosiphon pisum*)
c16514	1.85	cuticular protein 52 (*Acyrthosiphon pisum*)
c65979	1.99	RR1 cuticle protein 11 (*Acyrthosiphon pisum*)
c46947	3.63	Cuticle protein 6 (*Blaberus craniifer*)
c36838	6.08	RR1 cuticle protein 6 (*Acyrthosiphon pisum*)
c53043	6.63	structural constituent of cuticle
c66065	−2.25	cuticular protein 11 precursor (*Acyrthosiphon pisum*)
c78688	−1.57	cuticular protein 62 precursor (*Acyrthosiphon pisum*)
**Ribosomal Protein-Related Genes**		
c81455	1.12	large subunit ribosomal protein 6 (*Plasmodium berghei*)
c5335	1.20	ribosomal protein L10Ae-like (*Acyrthosiphon pisum*)
c11420	1.26	28S ribosomal protein S18b (*Acyrthosiphon pisum*)
c69386	1.34	39S ribosomal protein L35 (*Acyrthosiphon pisum*)
c91354	1.74	40S ribosomal protein S21-like (*Acyrthosiphon pisum*)
c75408	1.83	Ribosomal protein L15
c14330	1.94	ribosomal protein L27a (*Acyrthosiphon pisum*)
c27431	2.49	28S ribosomal protein S2 (*Acyrthosiphon pisum*)
c127488	4.8	28S ribosomal protein S29 (*Acyrthosiphon pisum*)
c31733	−1.40	ribosomal protein S27-1 (*Acyrthosiphon pisum*)
c116976	−1.36	ribosomal protein L41 (*Drosophila melanogaster*)
c51305	−1.25	39S ribosomal protein L41 (*Acyrthosiphon pisum*)
c31252	−1.09	40S ribosomal protein S7-like (*Acyrthosiphon pisum*)

**Table 3:** Some of the important differentially accumulated proteins and their relative log_2_ ratio between virus-infected and healthy *M. persicae* adults.

r	Accession Number	Name	log_2_ Ratio
Up-regulated	E5LMN6	Cytochrome b (fragment)	0.75
	Q1ZZP7	Cytochrome B5-like protein	0.85
	J9JMZ2	NADPH--cytochrome P450 reductase	1.47
	V5SQ25	Cytochrome P450 CYP6CY3	1.51
	J9K284	Ribosomal protein L15	1.54
	Q9TFD9	Cytochrome c oxidase subunit 2 (fragment)	1.74
	J9K6M4	Calcium-transporting ATPase	2.36
Down-regulated	Q45V96	Tentative cuticle protein	−1.64
	J9JYX3	ATP synthase subunit alpha	−1.25
	J9KB74	Glutamate dehydrogenase	−1.22
	Q1W9N4	Putative heat shock protein hslU	−0.81
	Q8VUS1	Chaperone protein DnaK (fragment)	−0.78
	Q45V97	RR2 cuticle protein 3 (fragment)	−0.76
	B5LYP1	Juvenile hormone binding protein	−0.74
	Q45V95	Cuticle protein 4	−0.62

**Table 4:** Significantly enriched KEGG pathways in the *M. persicae* proteome for aphids infected with cucumber mosaic virus.

Pathway	Number of Proteins		*p*-Value	ID
	DAPs ^†^	Accumulated ^‡^		
Valine, leucine, and isoleucine degradation	13	25	0.000235925	api00280
Fatty acid metabolism	16	42	0.000751229	api01212
Alanine, aspartate, and glutamate metabolism	9	15	0.001029258	api00250
Arginine and proline metabolism	11	25	0.0020428	api00330
Fatty acid degradation	10	21	0.002064943	api00071
Propanoate metabolism	6	11	0.010188421	api00640
Fatty acid biosynthesis	6	11	0.010188421	api00061
beta-Alanine metabolism	6	13	0.018211309	api00410
Proteasome	11	38	0.024134177	api03050
Spliceosome	22	100	0.025505972	api03040
Carbon metabolism	17	73	0.030987964	api01200
Biosynthesis of amino acids	12	47	0.038879652	api01230
Pyruvate metabolism	8	26	0.039544834	api00620
Nitrogen metabolism	4	8	0.043309293	api00910

^†^ The number of differentially accumulated proteins (DAP) that belong to each KEGG pathway. ^‡^ The number of accumulated proteins that belong to each KEGG pathway.

**Table 5:** Correlation between mRNA expression and the corresponding protein levels for 22 DEGs in CMV-infected adult *M. persicae*.

Gene ID	Protein ID	Log_2_ (Transcript Ratio)	Log_2_ (Protein Ratio)	E-Value	Annotation
c118109	V5SQ25	1.769388711	1.506921778	2.00 × 10^−69^	Cytochrome P450 CYP6CY3 (*Myzus persicae*)
c49464	Q9TFD9	6.480679524	1.733540171	2.00 × 10^−51^	Cytochrome c oxidase subunit 2 (fragment)
c52418	J9K5U3	1.211651723	1.824611319	7.00 × 10^−61^	Ubiquitin carboxyl-terminal hydrolase
c75408	J9K284	1.832888752	1.5354622	3.00 × 10^−28^	Ribosomal protein L15
c45978	J9K6M4	1.010053019	2.360933037	1.00 × 10^−6^	Calcium-transporting ATPase
c38643	J9K3N8	7.708471879	1.181065157	2.00 × 10^−4^	Coatomer subunit alpha
c25651	X1WJB6	9.50581	1.33764	3.00 × 10^−52^	-
c35347	Q64F38	−1.36053284	−1.74027779	1.00 × 10^−4^	Tropomyosin (*Myzus persicae*)
c11315	J9JYX3	−1.21150443	−1.23817987	6.00 × 10^−6^	ATP synthase subunit alpha
c41875	J9K071	−3.07923	−1.09732	3.00 × 10^−7^	-
c34089	Q45V96	0.4009223	−1.6241371	1 × 10^−10^	Tentative cuticle protein (*Myzus persicae*)
c30589	X1WIC4	0.4215968	1.2683861	0	Signal recognition particle subunit SRP72
c49978	J9KB74	0.1445756	−1.2304454	0	Glutamate dehydrogenase (*Acyrthosiphon pisum*)
c13794	J9JM35	−0.0390524	1.0775299	0	Amino acid transporter
c41961	J9JMR5	0.046467	1.1028535	1 × 10^−120^	Protein BCCIP homolog
c51904	J9JMZ2	−0.0417784	1.4710292	0	NADPH-cytochrome P450 reductase
c53037	X1XJB2	0.253985	−1.3529881	2 × 10^−52^	Histone H2B (fragment)
c52517	J9JWC3	0.0351009	2.2677165	0	Eukaryotic translation initiation factor 3 subunit E (*Acyrthosiphon pisum*)
c28628	J9KB65	−0.0646973	2.1664679	0	Lipase maturation factor
c49848	Q8B4P7	1.6937694	−1.2378697	4 × 10^−89^	Putative structural protein
c36095	J9K3Z0	−1.7250354	1.8737054	0	AP-3 complex subunit delta
c53987	J9JRR5	−1.1789701	1.6216423	0	-

## Data Availability

The data presented in this study are available in article and Supplementary Materials.

## Supplementary Materials

The following are available online at https://www.mdpi.com/article/10.3390/insects12050372/s1. Table S1. Primers used for qRT-PCR analyses, Table S2. Differentially-accumulated genes for the transcriptome dataset, Table S3. The most and highly and lowest DEGs, Table S4. GO-enriched for transcriptome dataset, Table S5. KEGG pathway for transcriptome dataset, Table S6. qRT-PCR verified differentially expressed genes, Table S7. Identification of differentially-accumulated proteins in Unitprot database, Table S8. Integrated function annotation for proteomic dataset, Table S9. Significant changes in both the transcript and the protein levels.
